# Supplementary material for: Timely initiation of antenatal care and its associated factors among pregnant women in sub-Saharan Africa: A multicountry analysis of Demographic and Health Surveys
Source: PLoS One. 2022 Jan 10;17(1):e0262411. doi: 10.1371/journal.pone.0262411 (PMC8746770; doi:10.1371/journal.pone.0262411)
Supplement: Attachment — Submitted filename: PONE-D-20-36739_revisions.docx [file pone.0262411.s001.docx]

PONE-D-20-36739

Timely initiation of antenatal care and its associated factors among pregnant women in

Sub-Saharan Africa: A multicounty analysis of Demographic and Health Surveys.

**Abstract**

**Background**

1. *“Mothers who attend ANC timely are fully benefited from its preventive and curative services.”*

change to:

Mothers who begin attending ANC in a timely manner, can fully benefit from preventive and curative services.

**Results**

2. please use a consistent number of decimals throughout the paper. E.g.,: 37.99% (95% CI: 37.79-38.19), ranging from 14.5**0**% in Mozambique to 68.6**0**% in Liberia.
3. “women from the community with medium education (AOR = 1.45; 95%CI: 1.35, 1.51), and women from the community with high education (AOR = 1.50; 95%CI: 1.33, 1.68) “

Not clear what women from the community with medium education? Do you mean women living in communities in the 2^nd^ tertile of education for example?

4. “However, women who wanted their pregnancy later (AOR = 0.84; 95%CI: 0.82 ,0.86), wanted no more (AOR = 0.80; 95%CI: 0.77, 0.83)”

I think there is a word missing after no more…

**Conclusion:**

5. SSA recorded a lower overall prevalence of timely initiation of ANC. Please change to:

SSA recorded a low overall prevalence of timely initiation of ANC.

6. “community women’s education”: please reword this is not clear.
7. “Therefore, more effort should be done to improve women’s education, wealth status of a household and family planning to prevent unwanted pregnancy and and then to improve the timely initiation of ANC.” This conclusion needs to be revised. Improving women’s wealth status is not an actionable strategy for public health practicioners. Please revise.

**Background section**

8. The background addresses maternal mortality at length. However, antenatal care does not have a strong relation to maternal deaths. Rather, it is quality intrapartum care that more strongly determines whether the mother survives. Quality ANC however, can have an important effect of fetal health and neonatal survival. Could the authors revise the background section to address the importance of ANC with more nuance, commenting on the importance of ANC to prevent miscarriages and stillbirths among others.

Please also remove the sentence: “ANC is the most important determinant of pregnancy outcomes”

9. The sentence: “85% of mothers start their ANC follow up earlier compared to below 45%...” is not clear. Do you mean that 85% start in the first trimester? Please revise.

**Methods**

10. Can you please describe how you selected these 36 surveys? Did you have a cut off date? Did you include all SSA countries?
11. Please change “ever had of a terminated pregnancy” to ever had a pregnancy terminated
12. Please change working status to employment status
13. perception of distance from the health facility (big problem/not a big problem) is not a community level factor. It is an individual level factor that is influenced by the woman’s ability to travel.
14. Please explain how you calculated community-level wealth. The following sentence is confusing: the proportion of women in the poorest and poorer quantiles in the community. What are “poorer” quintiles? 1 to 4?

**Statistical analysis**

15. The multi-level model is not clear. The authors should describe whether they used a random intercept or random slope model. and what level of clustering was used? The PSU, region or the country?
16. The first model (empty model) should contain the random effect. Is that what the authors did? Please revise the description.
17. Can you explain what deviance is? And why is model III in brackets after this sentence?

“To select the best-fitted model deviance was used and the

model with the lowest deviance was selected (model III).”

18. Please explain how you obtained the ICC (also known as variance partition coefficient in a logistic model)
19. Please describe what you mean by Median Odds Ratio (and include a reference for readers not familiar with the term).
20. I don’t see any benefit to running model 2 separately (community level variables only). I would recommend model 1: empty model, model 2: model with individual variables only and finally full model 3 with individual and community variables. The authors can then comment on the change in community-level variable as variables are added in. please cite: *Intermediate and advanced topics in multilevel logistic regression analysis Peter C. Austin and Juan Merlo*

**Results**

21. How did you pool results across countries? DHS sampling weights are not meant for multi-country comparisons. Each country should either be weighted equally or should be re-weighted based on population size for example. Another option would be to list result as: exposure to media ranged from X% in Botswana to X% in Rwanda.
22. Same comment for the regression models. Did you include sampling weights? Please also explain whether you pooled all the data together or ran models separately in every country…
23. This doesn’t seem correct: Most participants had attained higher education (n = 9,335, 40.0%). According to a quick internet search: only 6 **percent** of people in sub-Saharan **Africa** are enrolled in higher **education** institutions compared to the global average of 26 **percent**. Source: <http://www.aaionline.org/wp-content/uploads/2015/09/AAI-SOE-report-2015-final.pdf>
24. Replace three-fourth to three quarters
25. **Table 2: According to table 2 it is 4% that have achieved higher educaiton. Not 40%. Please review table 2 and make sure all calculations are correct.**
26. Community women’s education: in the table is described as low, medium, high. However, this is simply the % of women in the community who have achieved primary school. This is misleading, as primary education is not a “high” education achievement. Therefore I would recommend replacing with first, second and third tertile. Or by the actual range of % of women who achieved primary school e.g., <15%, 15%-40%, >40%.
27. Prevalence of timely initiation of first ANC visit in SSA: replace counties to countries.
28. Figure 1: please rank the countries by timely ANC initiation.
29. In table 3, please insert” reference” for PCV under the 1^st^ model.

**Discussion**

30. The conclusions and policy implications need substantial improvement. Improving women’s wealth status is not an actionable strategy for public health practitioners. Please remove this recommendation. The authors should discuss what can be done to promote earlier access to antenatal care in SSA and the kinds of strategies to target poorer/less educated women and their communities, more at risk to starting ANC late. There must be literature on this. And others must have tested strategies to achieve this.
31. In the background and discussion sections, please also expand on the benefits for mothers, but particularly newborns, of starting ANC in the first trimester of gestation. For example, detecting and treating different infections (e.g., malaria, syphilis, HIV etc.) early in the pregnancy can have substantial effects on improving fetal and newborn outcomes. Providing nutritional supplements and vitamins can also improve fetal and newborn outcomes. E.g., folic acid in the first trimester is crucial.
32. Please add a paragraph on the need for ANC to be of good quality in order to have any effect. Poor quality ANC, even if started early, is unlikely to improve health outcomes. There are many references on quality of antenatal care and inequities in ANC access and quality.

Other references possibly of interest: <https://www.ncbi.nlm.nih.gov/pmc/articles/PMC7457794/#:~:text=Parity%2C%20number%20of%20alive%20children,pregnancy%20approval%20by%20a%20spouse>.

<https://bmcpregnancychildbirth.biomedcentral.com/articles/10.1186/1471-2393-14-287>

<https://www.hindawi.com/journals/aph/2017/1624245/>

<https://journals.plos.org/plosone/article?id=10.1371/journal.pone.0246230>
